# New insights into the molecular mechanisms of ROR1, ROR2, and PTK7 signaling from the proteomics and pharmacological modulation of ROR1 interactome

**DOI:** 10.1007/s00018-022-04301-6

**Published:** 2022-05-04

**Authors:** Juuli Raivola, Alice Dini, Kari Salokas, Hanna Karvonen, Wilhelmiina Niininen, Emilia Piki, Markku Varjosalo, Daniela Ungureanu

**Affiliations:** 1 Applied Tumor Genomics Research Program, Faculty of Medicine, University of Helsinki, 00014 Helsinki, Finland; 2 Institute of Biotechnology, HiLife, University of Helsinki, 00014 Helsinki, Finland; 3 Faculty of Medicine and Health Technology, Tampere University, 33014 Tampere, Finland; 4 Faculty of Biochemistry and Molecular Medicine, University of Oulu, 90014 Oulu, Finland; 5 Herbert Irving Comprehensive Cancer Center, Columbia University Irving Medical Center, New York, NY 10032 USA

**Keywords:** Receptor pseudokinases, Kinase inhibitors, GZD824, Wnt signaling, ROR1, ROR2, PTK7, TSA, CETSA

## Abstract

ROR1, ROR2, and PTK7 are Wnt ligand-binding members of the receptor tyrosine kinase family. Despite their lack of catalytic activity, these receptors regulate skeletal, cardiorespiratory, and neurological development during embryonic and fetal stages. However, their overexpression in adult tissue is strongly connected to tumor development and metastasis, suggesting a strong pharmacological potential for these molecules. Wnt5a ligand can activate these receptors, but lead to divergent signaling and functional outcomes through mechanisms that remain largely unknown. Here, we developed a cellular model by stably expressing ROR1, ROR2, and PTK7 in BaF3 cells that allowed us to readily investigate side-by-side their signaling capability and functional outcome. We applied proteomic profiling to BaF3 clones and identified distinctive roles for ROR1, ROR2, and PTK7 pseudokinases in modulating the expression of proteins involved in cytoskeleton dynamics, apoptotic, and metabolic signaling. Functionally, we show that ROR1 expression enhances cell survival and Wnt-mediated cell proliferation, while ROR2 and PTK7 expression is linked to cell migration. We also demonstrate that the distal C-terminal regions of ROR1 and ROR2 are required for receptors stability and downstream signaling. To probe the pharmacological modulation of ROR1 oncogenic signaling, we used affinity purification coupled to mass spectrometry (AP-MS) and proximity-dependent biotin identification (BioID) to map its interactome before and after binding of GZD824, a small molecule inhibitor previously shown to bind to the ROR1 pseudokinase domain. Our findings bring new insight into the molecular mechanisms of ROR1, ROR2, and PTK7, and highlight the therapeutic potential of targeting ROR1 with small molecule inhibitors binding to its vestigial ATP-binding site.

**Supplementary Information:**

The online version contains supplementary material available at 10.1007/s00018-022-04301-6.

## Introduction

Receptor tyrosine kinases (RTKs) are transmembrane proteins that relay growth-factor signals to sustain a diverse set of biological responses from cell proliferation to apoptosis and differentiation [1]. A distinct subfamily of RTKs comprises of Wnt receptors, such as receptor tyrosine kinase orphan-like receptor 1 and 2 (ROR1/2) and protein tyrosine kinase 7 (PTK7). These receptors are also considered to be pseudokinases due to their (pseudo)kinase domain sequence deviations from the canonical kinase domain motifs [2, 3]. Structural and biochemical analysis of their pseudokinase domains revealed a close resemblance to the autoinhibited kinase domain of the insulin receptor; however, these molecules are capable of modulating signaling through conformational dynamics [3].

Despite their status as “dead” enzymes, these Wnt-binding RTKs are dysregulated in multiple diseases such as cancer and developmental disorders. The oncogenic functions of these receptors can be unleashed through amplification and overexpression via transcriptionally regulated mechanisms, and elevated ROR1, ROR2, and PTK7 expression is linked to tumor development and metastasis in several hematological as well as solid malignancies [4, 5]. Binding of Wnt5a to ROR1 and/or ROR2 can induce receptor homo- or hetero-dimerization to activate the signaling of AKT/ERK, Rho GTPases, NF-κB, and STAT3 pathways resulting in cell survival, proliferation, differentiation, and migration [5, 6]. PTK7 signaling is also linked to cell migration via cytoskeleton reorganization and planar cell polarity (PCP) regulation [7–9]. Although some of the molecular mechanisms mediated by Wnts binding to ROR1/2 and PTK7 have been revealed, our current understanding of their signaling properties and therapeutic potential is in its infant stages compared to pathways like RAF-MEK-ERK. Targeting the extracellular region of these receptors has been successful for the development of monoclonal antibodies with anti-tumor properties [10–13]; however, small molecule inhibitors targeting their intracellular regions have been described only for ROR1 [3], largely because their structural and functional properties are not fully characterized. We identified ponatinib and GZD824 (Olverembatinib), both structurally related pan-Bcr-Abl tyrosine kinase inhibitors, capable to bind to ROR1 pseudokinase domain and modulate its downstream signaling [3].

Previously, we observed that the oncogenic signaling mediated by ROR1 expression in IL-3-dependent pro-B murine BaF3 cells could render growth-factor independent proliferation of BaF3-ROR1 clones, whereas the expression of ROR2 and PTK7 could prolong cell survival, but did not achieve oncogenic transformation [3]. However, the molecular mechanisms mediating these functional differences have not been characterized. Here, we used state-of-the-art quantitative proteomics to identify the intracellular networks activated by ROR1, ROR2, and PTK7 stable expression in BaF3 cells. Functionally, we show that ROR1 expression enhances cell survival and Wnt-mediated cell proliferation, whereas ROR2 and PTK7 expression promotes cell motility. Furthermore, we explored the functional role of the C-terminal regions of ROR1/2 in sustaining receptor stability and signaling activation and showed that binding of GZD824 to the ROR1 pseudokinase domain stabilizes its intracellular region. Finally, we performed AP-MS and BioID for mapping the protein–protein interaction (PPI) network of ROR1 before and after its pharmacological modulation using GZD824. Taken together, our data provide an in-depth analysis of ROR1, ROR2, and PTK7 signaling network and their functional properties, underscoring the oncogenic role of ROR1 and its therapeutic potential using small molecule inhibitors binding to its pseudokinase domain.

## Materials and methods

### Cell culture and transfections

BaF3 cells (DSMZ, #ACC 300) were grown in RPMI media (Lonza, Basel, Switzerland) supplemented with 10% FBS, 50 U/ml penicillin, 50 µg/ml streptomycin, and 10% WEHI conditioned supernatant. For stable expression of human ROR1, ROR2, and PTK7 (or their truncated variants) in BaF3, proteins were cloned into the pEF-IRES-P vector [14] and transfected using Nucleofection as previously described [3].

293T cells were cultured in DMEM media (Gibco™, Thermo Fisher Scientific, Waltham, MA, USA) supplemented with 10% FBS, 50 U/ml penicillin, 50 µg/ml streptomycin. Transient transfection of 293T cells was done using TurboFect (Thermo Fisher Scientific) according to the manufacturer’s instructions.

### Immunoblotting

Whole cell lysates were prepared and the immunoblotting was done as previously described [3] in Triton X 100-containing lysis buffer (50 mM Tris–HCl, pH 7.5, 10% glycerol, 150 mM NaCl, 1 mM EDTA, 1% Triton X-100, 50 mM NaF). For Western blotting, the following antibodies were used: pAKT (S473, #6942), AKT (#9272), Bcl-xL (#2764), pERK1/2 (#9101), MEK1/2 (#4694), pMEK1/2 (#9121), ERK1/2 (#4696), NF-κB p65 (#6956), pNF-κB p65 (#3033), PARP (#9532), PI3K p85α (#13,666), pPI3K p85/p55 (#4228), PTK7 (#25618), Rac-1 (#4651), RhoA (#2117), ROR1 (#16,540), ROR2 (#88,639), Src (#2109), STAT3 (#9139), pSTAT3 (#9145) from Cell Signaling Technology (CST, Danvers, MA, USA); anti-pTYR 4G10 (#05–321) from Merck Millipore (Burlington, MA, USA); β-tubulin (#sc-166729) from Santa Cruz Biotechnology (Dallas, TX, USA); HA (#901,513) from BioLegend (San Diego, CA, USA). As secondary antibodies, IRDye^®^ 800CW Donkey anti-Mouse IgG or IRDye^®^ 680RD Donkey anti-Rabbit IgG (LI-COR, Lincoln, NE, USA) were used at 1:10 000 dilution.

### Recombinant protein production

ROR1 proteins were cloned into the pFastBac1 vector with a C-terminal His_6_ tag and expressed in *Spodoptera frugiperda* (Sf9) cells using the Bac-to-Bac™ baculovirus expression system (Gibco™) according to the manufacturer’s instructions.

The constructs and their amino acid (aa) boundaries are listed in the table below.

**Table:** 

ID	Domain	aa
Δ1	PK	453–746
Δ2	PK-S/T	453–782
Δ3	PK-S/T-P	453–851
Δ4	PK-S/T-P-S/T	453–876
Δ5	FL-Cyto	453–937
JAK2	Pseudokinase domain	536–827
MuSk	Kinase domain	520–869

In brief, Sf9 cells were infected at 2 × 10^6^/ml with recombinant baculovirus and harvested by centrifugation after 3 days. Cells were lysed by sonication in Buffer A containing 20 mM Tris–HCl (pH 8.0), 300 mM NaCl, 10% (w/v) glycerol, 10 mM imidazole, 10 mM β-mercaptoethanol, 1 mM PMSF, and protease inhibitor cocktail (Roche, Basel, Switzerland), and the lysate was clarified by centrifugation for 30 min at 16 000 rpm at 4 °C. Lysates were mixed with pre-washed Ni–NTA beads (QIAGEN, Hilden, Germany) and rotated 1 h at 4 °C, after which they were again washed with 50 beads volumes of Buffer A. The bound ROR1 proteins were eluted with increasing concentrations of imidazole in Buffer B (Buffer A + 400 mM imidazole), and the eluted proteins were further purified with size exclusion chromatography using a Superdex 75 10/300 column (GE Healthcare, Chicago, IL, USA) equilibrated in 20 mM Tris (pH 8.0), 150 mM NaCl, and 2 mM DTT.

### Differential scanning fluorimetry (DSF)

For thermal shift assays to study ATP binding of the recombinant ROR1 domains, the proteins were diluted in 20 mM Tris (pH 8.0), 150 mM NaCl, and 100 μM TCEP to a final protein concentration of 5 μM. Nucleotides (5 mM) and/or cations (10 mM of MgCl_2,_ MnCl_2_ or CaCl_2_) were added. SYPRO Orange (Thermo Fisher Scientific) was added to make a 2 500-fold dilution into the sample, and 25 μl of the reaction mixtures were transferred to Concord 96-well polycarbonate PCR plates (Bio-Rad Laboratories, Hercules, CA, USA) with three technical replicates per condition. To monitor the fluorescence at 530 nm, a Bio-Rad CFX96 Touch Real Time PCR machine was used, where the temperature was raised by 1 °C per minute from 25 °C to 95 °C. The fluorescence was measured at each increment, and the values were plotted as a function of temperature after normalizing according to the maximum fluorescence signal. Melting temperatures (T_m_) were determined as the temperature at half-maximum fluorescence. Plots were generated using GraphPad Prism and represent the mean from three technical replicates.

### Cellular thermal shift assay (CETSA)

CETSA was carried out according to the protocol described in [3] and [15]. Briefly, BaF3-ROR1-expressing cells were treated with control (DMSO) or drug (10 μM) and incubated at 37 °C for 2 h. Cells were washed once and divided into aliquots with 0.5 × 10^6^ cells per sample. Samples were heated (Bio-Rad T100TM Thermal Cycler) pairwise (control and drug-treated samples), and after heating for 3 min at 42–60 °C with 2 °C increments between pairs, cells were collected and lysed in buffer supplemented with protease and phosphatase inhibitor cocktails (Bimake, Huston, TX, USA). The samples were subsequently analyzed by SDS-PAGE and immunoblotting using anti-HA (ROR1) and β-tubulin antibodies. Protein levels were quantified with Image Studio Lite (Li-COR) and normalized to 42 °C untreated samples.

### Proliferation assay

To measure the cell proliferation, Cell Titer-Glo (CTG) Luminescent Cell Viability Assay 2.0 (Promega, Madison, WI, USA) was used according to the provider’s instructions. Briefly, cells were plated onto 96-well plates and starved for 24 h, after which cells were treated with 100 ng/ml of recombinant Wnt5a or Wnt16b (R&D Systems, Minneapolis, MN, USA) or left untreated. After 24 h or 48 h, an equal volume of CTG reagent was added to each well and incubated for 30 min at room temperature before measuring the luminescence with Envision plate reader (Perkin-Elmer, Waltham, MA, USA).

### Migration assay

The migration assay was performed in HTS Transwell^®^-96-well plates (Corning Incorporated, Corning, NY, USA) with 5.0 µm pore size polycarbonate membranes following the manufacturer’s instructions. BaF3 cells were seeded in the Transwell upper insert and the lower insert contained media with 200 ng/ml of CXCL12 (R&D Systems, 350-NS-010), and were incubated for 6 h at 37 °C and 5% CO_2_ after which the cell number in the bottom chamber was counted by Accuri C6 Flow Cytometer (BD Biosciences, Franklin Lakes, NJ, USA). The migration of BaF3 clones was normalized to the values obtained from BaF3 parental cells and the statistical analysis (Student’s *t* test) was done in GraphPad Prism.

### DNA mutagenesis

ROR1 kinase-restoring mutations were done using Q5 site directed mutagenesis kit (New England Biolabs, Ipswich, MA, USA) according to the manufacturer’s instructions.

### Proteomic profiling via liquid chromatography–mass spectrometry (LC–MS/MS) data analysis

BaF3-ROR1, BaF3-ROR2, BaF3-PTK7, and BaF3 cells were cultured in four replicates and cell pellets were collected separately. The total protein concentration of samples was measured with Bio-Rad DC protein assay, and 50 µg of protein was precipitated with acetone (− 20 °C) overnight. Sample preparations for SWATH injection and proteomic analysis by Nano-RPLC-MSTOF instrumentation using Eksigent 425 NanoLC coupled to high-speed TripleTOF™ 5600 + mass spectrometer (Ab Sciex, Concord, Canada) were done at the Tampere Mass Spectrometry Facility of Tampere University. The data were normalized according to central tendency global median. Four biological replicates were kept for each sample, among which pairwise Pearson correlation was calculated (Fig. S1b-d) Principal component analysis (PCA) was performed on the scaled and centered data. Differentially expressed proteins (DEPs) were obtained with *limma* 3.50.0 [16] and the *p* values were adjusted according to Benjamini and Hochberg [17]. Gene ontology enrichment analysis was performed for each BaF3 clone’s significant DEPs with *GOfuncR* version 1.14.0 [18]. The GO categories were tested for enrichment with the function *go_enrich* using the hypergeometric test and the *Mus Musculus* organism annotation package 1.3.1 [19]. Next, the results were further processed with the function refine adopting a family-wise error rate (FWER) threshold of 0.05. Finally, only the GO biological process terms not including the words splicing, spliceosome, ribosomal, RNA, spliceosomal, snRNP, ribosome, and ribonucleoprotein were kept. The semantic similarity matrix was obtained with the R package *rrvgo* 1.6.0 [20]: first, the union of the retained GO biological processes terms from each BaF3 clone analyses was calculated, and the score similarity matrix was calculated according to the Wang distance with the function *calculateSimMatrix*. Then, the set of GO terms was reduced according to their semantic similarity with *reduceSimMatrix*.

### BioID and AP-MS samples’ preparation

#### Cell culture

The ROR1 gateway entry clone was obtained from a previously published collection [21]. This was then cloned into MAC-tag-C expression vector [22] via gateway cloning. For the generation of stable cell lines inducibly expressing the MAC-tagged ROR1, Flp-In 293 T-REx cell lines [cultured in DMEM (4.5 g/l glucose, 2 mM L-glutamine) supplemented with 50 mg/ml penicillin, 50 mg/ml streptomycin and 10% FBS] were co-transfected with RTK (Receptor Tyrosine Kinase) expression vector, and pOG44 vector (Invitrogen, Waltham, MA, USA) using FuGENE 6 transfection reagent. Cell lines were obtained directly from commercial sources; in addition, only low passage cells (passage number < 10) were used for experiments. Manufacturers are known to follow the authentication of cell line batches regularly, and certificates of authentication were provided with the cells, and checked before their use. Two days after transfection, selection was performed with 50 μg/ml streptomycin and 100 μg/ml hygromycin for 2 weeks, after which the positive clones were pooled and amplified. The cells were equally divided for treatment with 1 µM of GZD824 (for 1 h), or equivalent amount of water. For each treatment, half were then used for the AP-MS approach and the same amount for BioID. For AP-MS, expression of the bait protein was induced with 1 μg/ml tetracycline 24 h prior to harvesting to produce expression levels corresponding to the milder overexpression. With BioID, 50 μM biotin was added to the plates in addition to induction with tetracycline. Pervanadate was added at a concentration of 100 µM for 15 min prior to harvesting. Cells (~ 5 × 10^7^) were harvested on ice and pelleted, so that each bait protein had two biological replicates for each of the two approaches. Samples were snap frozen and stored at – 80 °C.

#### Affinity purification (AP)

After harvesting, samples were assigned a running identifier number for blinding instead of the gene/protein name of each bait. The name was then restored only once the data analysis began.

#### LC–MS/MS following BioID and AP

AP-MS and BioID purifications were carried out as outlined in the MAC-tag workflow publication [23] and as previously described [24]. The LC–MS/MS analysis was performed on Q-Exactive or Orbitrap Elite mass spectrometers using Xcalibur version 3.0.63 with an EASY-nLC 1000 system attached via electrospray ionization sprayer (Thermo Fisher Scientific) as previously described [24]. For each sample, two biological replicates were used. The Acquired MS2 spectral data files (Thermo RAW) were searched with Proteome Discoverer 1.4 (Thermo Scientific) as described in [24]. SEQUEST search engine was against human protein database extracted from UniProtKB (https://uniprot.org) on 15.03.2021, after which the data filtering steps were conducted as in [24] to identify the specific high-confidence interactors from AP-MS and BioID data.

#### Data analysis of the BioID and AP-MS samples

The respective protein–protein interaction (PPI) networks and their enrichment *p* values were obtained with STRING retaining only the physically interacting high-confidence interactors with interaction score > 0.700 and excluding the disconnected nodes from the network. The bar graphs showing the enriched ontology clusters ROR1 interactomes were extracted using Metascape [25]. AP-MS and BioID filtered data were processed using the ProHits-viz tools [26] (with the default settings) to carry out dot plot analyses displaying the protein interaction data.

## Results

### Proteomic profiling reveals common and distinct signaling landscape following ROR1, ROR2, and PTK7 expression

**Fig. 1:**
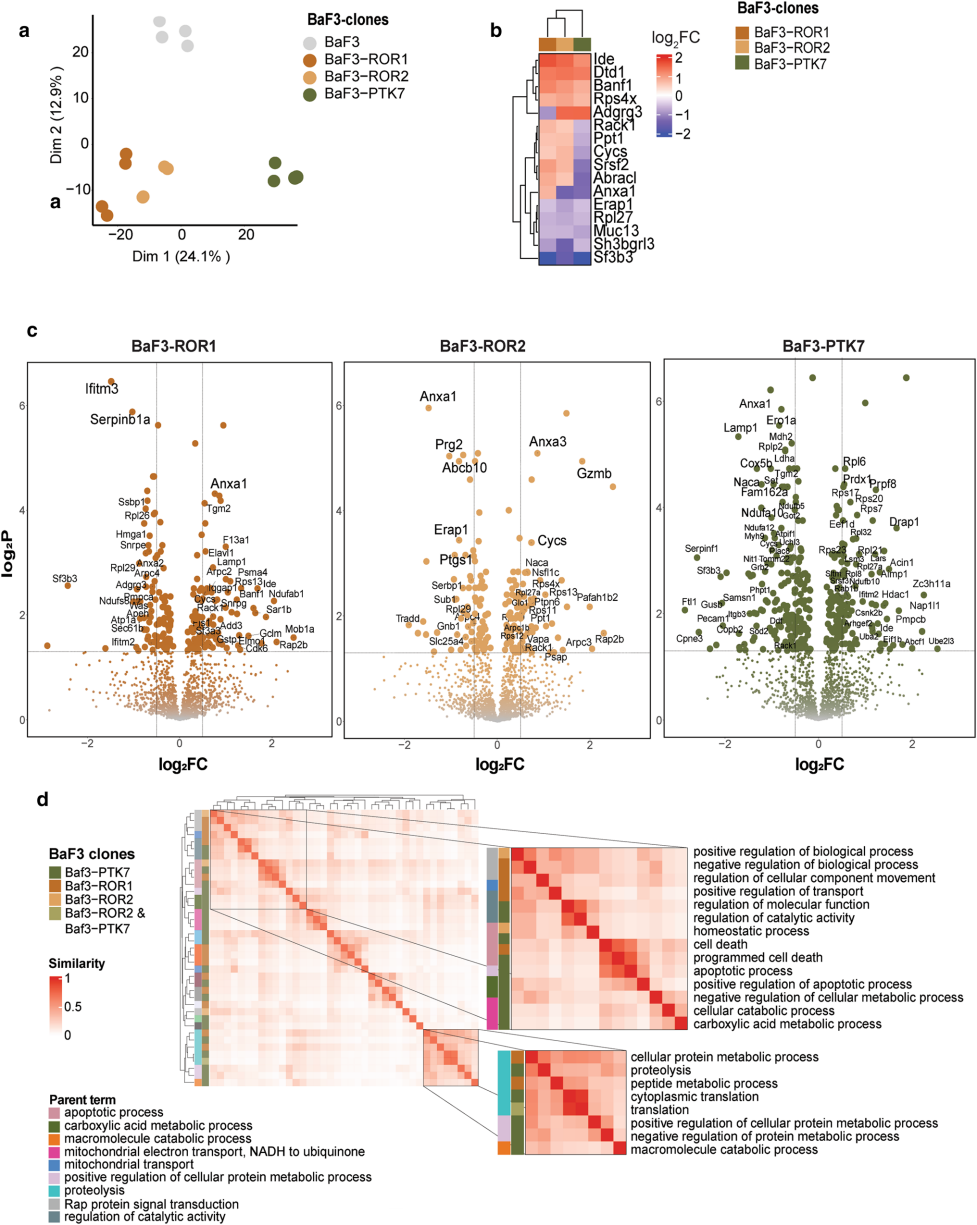
Proteomics analysis of ROR1, ROR2, and PTK7 transfected BaF3 cells. **a** Principal component analysis (PCA) showing phenotypic similarity between BaF3-ROR1 and BaF3-ROR2 biological replicates, but not among the parental BaF3 and BaF3-PTK7 clones. **b** Heatmap of differentially expressed proteins (DEP) log_2_ fold change with respect to the parental BaF3 samples. The DEPs shared among BaF3-ROR1, -ROR2, and -PTK7 are shown. **c** Volcano plots reporting the log_2_ fold change of the DEPs between the parental BaF3 cells and BaF3 clones. Adjusted* p* value < 0.05 and log_2_FC >|0.5|. **d** Similarity matrix (shown as a heatmap) among GO biological processes related to DEPs of each BaF3 clone. The apoptotic- and metabolic-related processes are highlighted. The heatmaps in **b** and **d** are clustered using complete-linkage hierarchical clustering based on Euclidean distances

To gain insights into the signaling network mediated by Wnt-binding RTKs, we created stable clones expressing ROR1, ROR2, or PTK7 in IL-3-dependent BaF3 cell line, which otherwise lacks endogenous expression of these receptors (Fig. S1a). To identify differentially regulated proteins by ROR1, ROR2, or PTK7 expression, we performed LC–MS/MS-based quantitative proteomic analysis of BaF3 clones and compared their proteomic profile with the parental BaF3 cells. Principal component analysis (PCA) showed that BaF3-ROR1 and BaF3-ROR2 clones map close to each other, but are significantly separated from BaF3-PTK7 clones and BaF3 parental cells, suggesting that BaF3-ROR1 and BaF3-ROR2 proteomic profiles are closely interrelated, as expected (Fig. 1a).

Comparison of differentially expressed proteins (DEPs, log_2_ fold change, *p* < 0.05) of each BaF3-ROR1, -ROR2, and -PTK7 clones with the parental BaF3 cells identified 16 proteins that were up- or down-regulated in all BaF3 clones, representing molecular effectors from metabolic to actin-cytoskeleton modulators (Fig. 1b, Table S1). Among commonly upregulated DEPs were insulin-degrading enzyme (Ide), D-aminoacyl-tRNA Deacylase 1 (Dtd1), barrier-to-auto integration factor (Banf1), and ribosomal protein S4 X-linked (Rps4x), whereas downregulation of endoplasmic reticulum aminopeptidase 1 (Erap1), receptor like protein 27 (Rlp27), mucin 13 (Muc13), and splicing factor 3B subunit 3 (Sf3b3) was observed in all BaF3 clones. Interestingly, receptor of activated protein C kinase 1 (Rack1), protein-tyrosine-phosphatase PTP1 (Ptp1), cytochrome c (Cycs), and costars family protein ABRACL (Abracl) upregulation was detected only in BaF3-ROR1 and BaF3-ROR2 clones, whereas Adhesion G protein-coupled receptor G3 (Adgrg3) upregulation was identified in BaF3-ROR2 and BaF3-PTK7 clones.

Several proteins from the family of actin related protein 2/3 complex (Arpc) were also significantly altered in the BaF3-ROR1 and BaF3-ROR2 clones (Fig. 1c). The expression of Arpc subunit 1b (Arp1b) and Arpc2 was increased in BaF3-ROR1, while Arpc3 was upregulated in BaF3-ROR2 cells. These proteins drive the Arp2/3 complex-mediated actin nucleation and cell migration and their subunits have been associated with cancer metastasis [27, 28]. On the other hand, upregulation of apoptotic chromatin condensation inducer1 (Acin1) and ribosomal protein 7 (Rsp7) in BaF3-PTK7 clones could suggest that PTK7 expression is involved in the modulation of apoptotic processes [29, 30].

To better understand the biological function of DEPs, we performed GO-enrichment analysis followed by semantic similarity calculation to uncover the several altered yet related biological processes. BaF3-ROR1 clones enriched with terms related to regulation of cellular component movement, cellular protein metabolic process, and positive regulation of transport, whereas BaF3-PTK7 clones were enriched for terms related to programmed cell death and apoptosis and regulation of cellular metabolic and catabolic processes (Fig. 1d, Table S2). BaF3-ROR2 clones were enriched only for positive regulation of biological processes and homeostasis (Fig. 1d).

### ROR1 expression supports pro-survival signaling, while ROR2 and PTK7 expression enhances cell motility

**Fig. 2:**
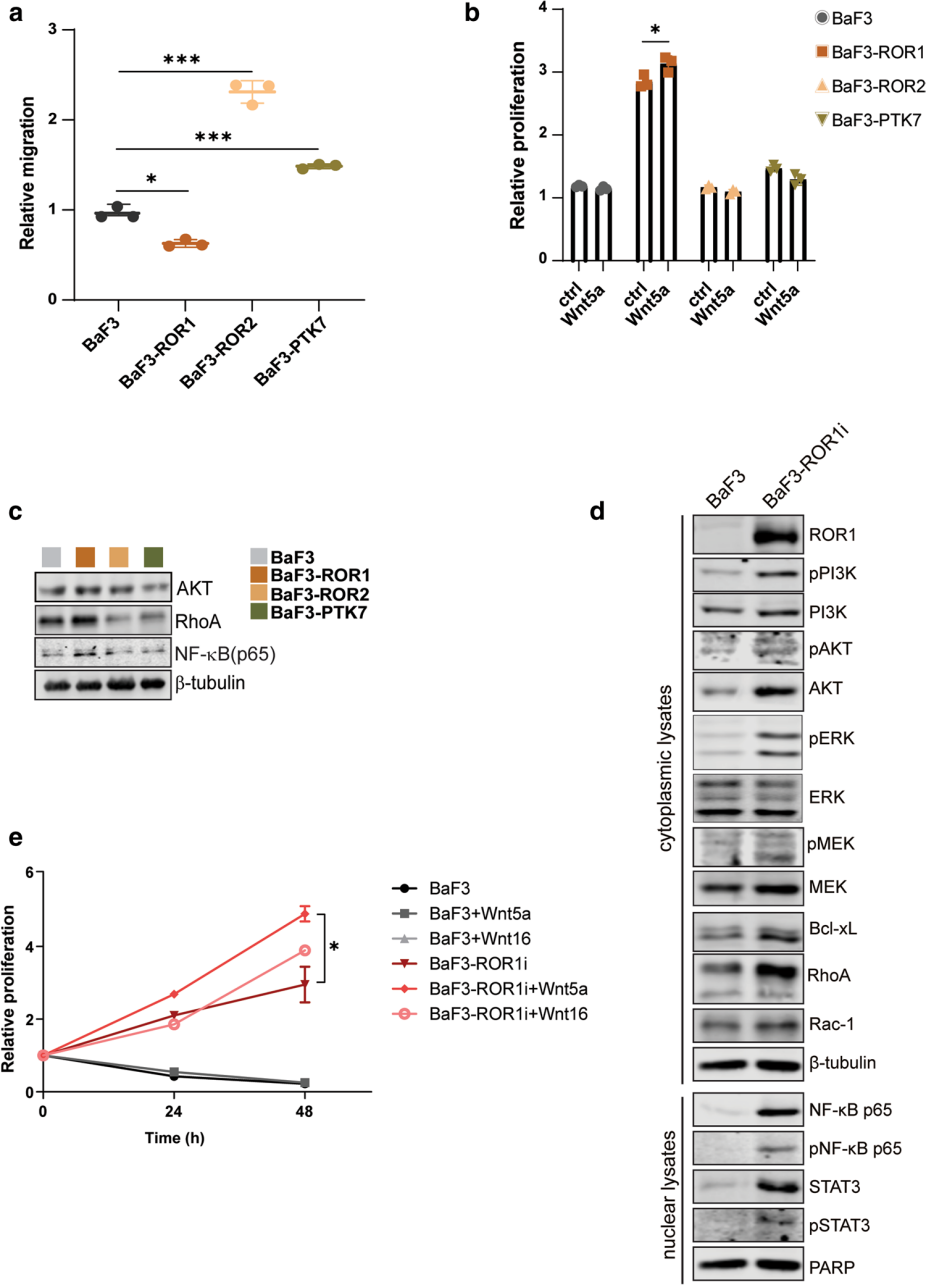
Functional analysis of BaF3-ROR1, BaF3-ROR2, and BaF3-PTK7 clones. **a** Migration assay of BaF3 clones toward CXCL12 chemokine for 6 h. The graph shows the relative number of migrated cells normalized to BaF3 parental migrated cells. Data are represented as mean ± standard deviation (SD) and statistical comparison was performed with Student’s *t* test (*n* = 3). **b** Relative survival of BaF3 cells cultured in starvation media (no serum and no IL-3) in the presence or absence of Wnt5a (100 ng/ml) for 24 h. The graph shows mean ± SD (*n* = 3). In a and b, the statistical significance is indicated as *p < 0.05 and ***p < 0.001. **c** Immunoblotting analysis of the intracellular signaling in BaF3 parental cells and BaF3 clones expressing ROR1, ROR2, or PTK7. β-tubulin was used as a loading control. **d** Immunoblotting analysis of the cytoplasmic and nuclear lysates of BaF3-ROR1i clones that acquired IL-3 independence. Antibodies against the effectors of the pro-survival signaling were used to detect the protein levels and β-tubulin was used as a loading control for the cytoplasmic lysates, whereas PARP was used as a loading control for the nuclear lysates. **e** The relative proliferation of BaF3 parental and BaF3-ROR1i clone stimulated with 100 ng/ml Wnt5a or Wnt16b. Proliferation was measured at the indicated time points and the values were normalized to non-stimulated cells (set as value 1). The graph shows the mean ± SD (*n* = 3). Statistical comparison between the non-stimulated and Wnt-stimulated BaF3-ROR1i cells was done using Student’s *t* test as *p < 0.05

Next, we interrogated the functional properties of BaF3 clones such as cell migration, survival, and Wnt-mediated signaling properties. We assessed the short-term migration potential using CXCL12 chemokine, which has been previously used to investigate lymphocytes chemotaxis [31]. An enhanced migration toward CXCL12 chemokine was observed for BaF3-ROR2 and BaF3-PTK7 but not BaF3-ROR1 clones when compared to BaF3 cells (Fig. 2a). However, BaF3-ROR1 clones showed the highest survival rate compared to other BaF3 cells in the absence of serum and IL-3, and this effect was modestly increased by the addition of recombinant Wnt5a (Fig. 2b). Moreover, immunoblotting analysis of BaF3 cell lysates showed higher levels of AKT, NF-κB and RhoA in BaF3-ROR1 clone compared to other BaF3 cells (Fig. 2c).

We have previously shown that BaF3-ROR1 clone could acquire growth-factor independence and proliferate in the absence of IL-3 [3] (named BaF3-ROR1i). Here, we investigated the activation of intracellular signaling in BaF3-ROR1i cells that were grown in the absence of IL-3. Immunoblotting analysis of cytoplasmic and nuclear lysates identified elevated PI3K/AKT/ERK, Bcl-XL, RhoA, STAT3, and NF-κB levels in BaF3-ROR1i compared to BaF3 parental cells (Fig. 2d). Furthermore, enhanced cell proliferation of BaF3-ROR1i cells was observed in the presence of recombinant Wnt5a and Wnt16 ligands, which were previously shown to bind to ROR1 [32, 33].

Taken together, our results demonstrate that ROR1 expression leads to activation of intracellular signaling such as PI3K/AKT/ERK, NF-κB, and STAT3, and these pathways could sustain BaF3 cell survival and Wnt-mediated proliferation.

### The C-terminal cytoplasmic region of ROR1 and ROR2 modulates signaling activation

**Fig. 3:**
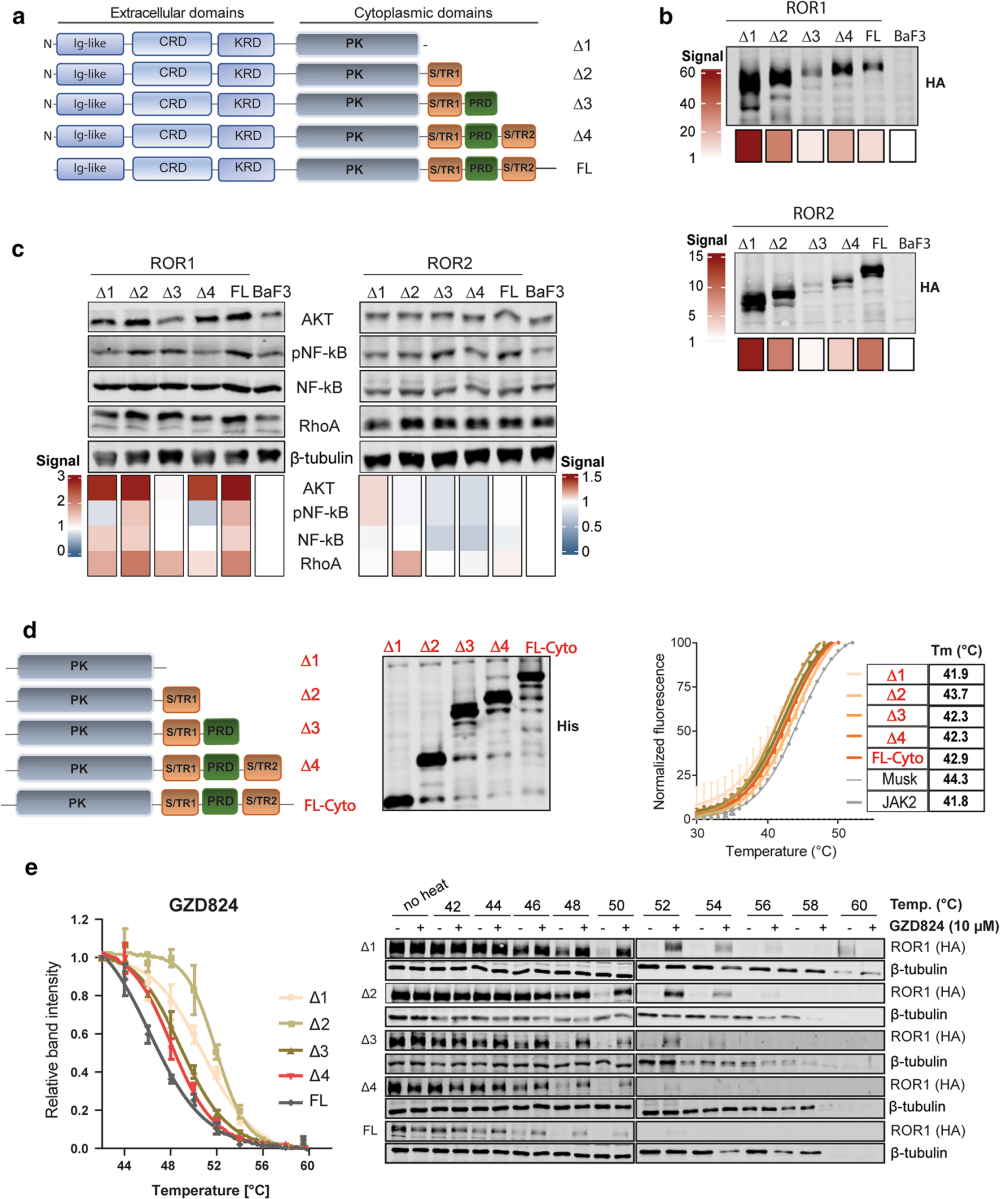
Characterization of the C-terminal cytoplasmic region of ROR1 and ROR2. **a** Schematic representation of amino acid boundaries of ROR1 and ROR2 C-terminal deletions (Δ1–Δ4) and the full-length (FL) receptors stably transfected into BaF3 cells. **b** Immunoblot analysis of the HA-tagged ROR1 and ROR2 FL and Δ1–Δ4 deletions expressed in BaF3 cells cultured for 24 h in starvation media. β-tubulin was used as a loading control and the signal quantifications shows the normalized values using BaF3 (set as 1). **c** Immunoblotting analysis of downstream signaling of BaF3-ROR1 and BaF3-ROR2 (FL and Δ1–Δ4) clones. β-tubulin was used as a loading control and signal quantifications show the normalized values using BaF3 samples. **d** Left: schematic representation of the cytoplasmic ROR1 constructs used for recombinant protein production. Expression levels of the His-tagged constructs were verified by immunoblotting. Right: DSF analysis of recombinant ROR1 constructs. Melting temperatures (T_m_) are also indicated, and the data represent the mean ± SD (*n* = 3). JAK2 PK domain and MuSK kinase domain was used as a reference control. **e** Cellular thermal shift assay (CETSA) showing stabilization of ROR1 (FL and Δ1–Δ4) upon treatment with GZD824 (10 µM). Cell lysates were heated to the indicated temperatures and immunoblotted with anti-HA for ROR1 detection. β-tubulin was used as a loading control. The graph shows the signal quantification of 3 independent experiments (mean ± SD). Band intensities were normalized to the 42 °C untreated samples for each ROR1 construct (set as 1)

Unlike PTK7, ROR1 and ROR2 cytoplasmic regions contain, in addition to a pseudokinase (PK) domain, two Ser/Thr-rich regions flanking a Pro-rich region, and a C-terminal tail (Fig. 3a). Most of the signaling initiation events were mapped to the Pro-rich and Ser/Thr-rich regions, which were attributed with key roles in binding/scaffolding molecules responsible for downstream ROR1/2 signaling [34–38]. To understand the importance of these distal regions in receptor signaling, we produced C-terminal ROR1 and ROR2 deletions (Δ1–Δ4) that were stably transfected in BaF3 cells. Analysis of ROR1 and ROR2 protein expression of BaF3 clones revealed that Δ3 deletion lacking the second Ser/Thr region and the C-terminal tail showed markedly lower expression when compared to full length (FL) for both receptors (Fig. 3b). These results were corroborated by similar findings obtained when the same ROR1 and ROR2 deletions were transiently transfected into 293T cells (Fig. S2a).

Next, we analyzed the downstream signaling activation of BaF3 clones stably expressing ROR1 and ROR2 C-terminal deletions by immunoblotting. ROR1 Δ3 deletion resulted in complete loss of AKT overexpression, whereas other ROR1 deletions had less impact (Fig. 3c). Surprisingly, the upregulated RhoA level following ROR1 FL expression in BaF3 cells was not affected by its C-terminal deletions, which could indicate different functions for ROR1 downstream molecules. On the other hand, we did not observe a clear effect of ROR2 C-terminal deletions on AKT, NF-kB or RhoA levels (Fig. 3c), which could indicate that downstream ROR2 signaling is different than ROR1 and we were not able to identify the pathways that are sensitive to ROR2 receptor truncations (Fig. 3c).

We then aimed to understand whether the C-terminal deletions would affect the stability of ROR1 cytoplasmic region only, possibly due to conformational changes in this region. For this purpose, we produced recombinant ROR1 proteins with the same deletions (Fig. 3d) and investigated their thermal stability using differential scanning fluorimetry (DSF). Overall, no major differences were observed in the melting temperature (T_m_) of ROR1 recombinant proteins representing Δ1–Δ4 deletions, suggesting that the functional relevance of the C-terminal region of ROR1 requires a FL receptor and is likely connected to its intracellular signaling machinery.

ROR1 PK domain contains several amino acid deviations from a canonical kinase domain motifs, which render ROR1 catalytically inactive, namely Gly to Cys in the glycine-rich domain, Arg to Lys in the His-Arg-Asp (HRD) motif, and Leu to Phe in the Asp-Phe-Gly (DFG) of the activation loop (Fig. S2c). Therefore, we sought to assess whether the restoration of glycine-rich and/or HRD and/or DFG motifs in ROR1 would affect its catalytic inactivity. Compared to a canonical active kinase such as Janus kinase 2 (JAK2), ROR1 wild-type or any of the kinase-restoring mutations did not result in significant auto-phosphorylation signal when overexpressed in 293 T cells (Fig. S2c). These results were further corroborated by the DSF analysis of ATP binding to recombinant ROR1 cytoplasmic proteins showing the absence of *T*_m_ shifts in the presence of ATP and different cations (Fig. S2d). Taken together, our results corroborate previous reports showing that ROR1 PK domain is unable to bind ATP and it can function in a catalytically inactive manner [2, 39].

Previously, we have shown that ROR1 PK domain can be targeted with small molecule inhibitors such as the tyrosine kinase inhibitors ponatinib or GZD824 [3], and binding of GZD824 to recombinant ROR1 PK domain induces strong concentration-dependent *T*_m_ shifts in DSF assay (Fig. S2d). We then assessed how GZD824 binding to ROR1 PK is modulated by the C-terminal regions using cellular thermal shift assay (CETSA) of BaF3-ROR1 clones [15]. GZD824 binding greatly stabilized Δ1 and Δ2 deletions compared to Δ3, Δ4, and FL ROR1 following heat denaturation (Fig. 3e), suggesting that these distal C-terminal regions affect the stability of the PK domain following drug binding.

### Characterization of ROR1 interactome using BioID and AP-MS

**Fig. 4:**
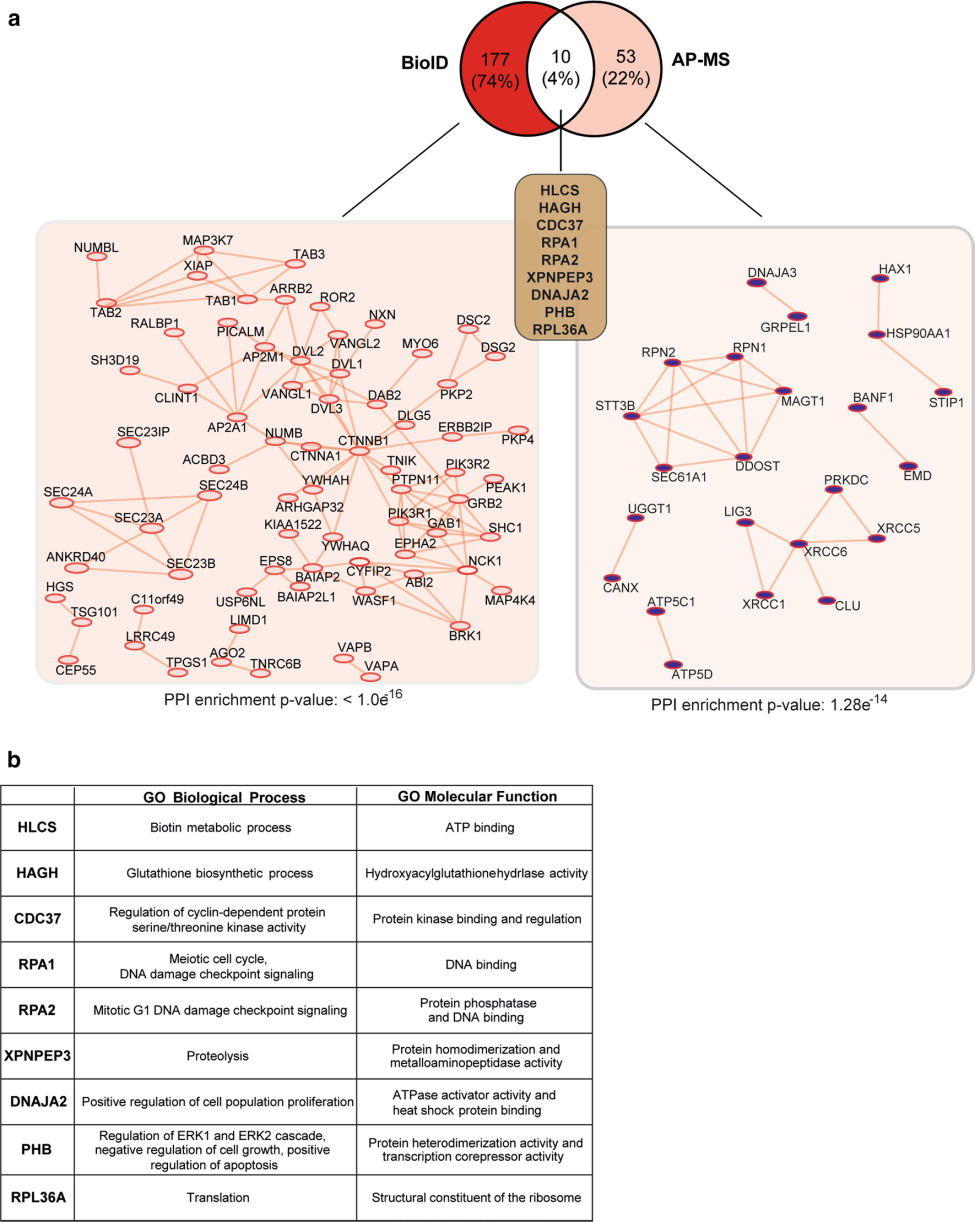
ROR1 interactome detected by BioID and AP-MS. **a** Venn diagram showing the number of specific ROR1 interactors identified with BioID (red), AP-MS (pink), or both (white). The respective physical interactomes and enrichment *p* values were obtained with STRING (reference as reported in the methods chapter). **b** List of selected GO biological processes and molecular functions associated with the ROR1 interactors and identified with both BioID and AP-MS

To gain better understanding of ROR1 signaling landscape mediating its pro-survival functions, we used two complementary methods, AP-MS and BioID [24] in an integrated approach combining both methods in a single construct termed Multiple Approaches Combined (MAC)-tag [22]. Following the transfection of MAC-tagged ROR1 into Flp-In T-REx 293 cells to establish transgenic, stably expressing, and inducible isogenic cell lines, the interacting proteins were analyzed by quantitative mass spectrometry where high-confidence interaction proteins were inferred via stringent statistical filtering. We detected a total of 240 interactors: 177 uniquely with BioID, 53 with AP-MS, and 10 with both (Fig. 4a, Table S3). The number of interactors identified with BioID was significantly higher compared to AP-MS, in accordance with the previous findings that BioID can detect proximal and transient interactions [22] while AP-MS allows the capture of more stable interactions [22]. Specifically, with BioID, we were able to map ROR1 to interaction modules related to cytoskeleton dynamics (ABI2, ROR2, PEAK1, and MYO6), MAPK signaling (ROR2, NOTCH, TAB1-3), Rho GTPase signaling (PI3KR1, PI3KR2), and non-canonical Wnt/planar cell polarity (PCP) pathway (VANGL1-2 and DVL1-3) (Fig. 4a left, Table S3).

Furthermore, among the proteins captured with AP-MS, we found HCLS1-associated protein X-1 (HAX1), which closely resembles cortactin and recruits the ARP2/3 complex to regulate actin-cytoskeleton reorganization [40]. Interestingly, Arpc subunit 1b (Arp1b) and Arpc2 levels were also increased in BaF3-ROR1 cells according to our proteomic analysis of DEPs. Moreover, ROR1 appeared to interact with STT3 oligosaccharyl-transferase STT3 Complex Catalytic Subunit B (STT3B), the catalytic subunit of oligosaccharyl-transferase (OST) complex, as well as with many of its accessory subunits (such as RPN1, RPN2, MAGT1, and DDOST) [41], which would indicate that ROR1 is involved in glycoproteome regulation.

Finally, the two approaches detected a common set of novel putative ROR1 interactors that have not been identified before. Among these proteins are stress-related protein chaperones Hsp90 co-chaperone (CDC37) and DNA J homolog subfamily A member 2 (DNAJA2) [42], DNA repair and replication proteins (RPA1 and RPA2), and prohibitin (PHB), a regulator of ERK signaling and marker of tumor aggressiveness in diffuse large B-cell lymphoma (Fig. 4b) [43, 44].

### Pharmacological targeting of ROR1 pseudokinase domain modulates its interactome and downstream functions

**Fig. 5:**
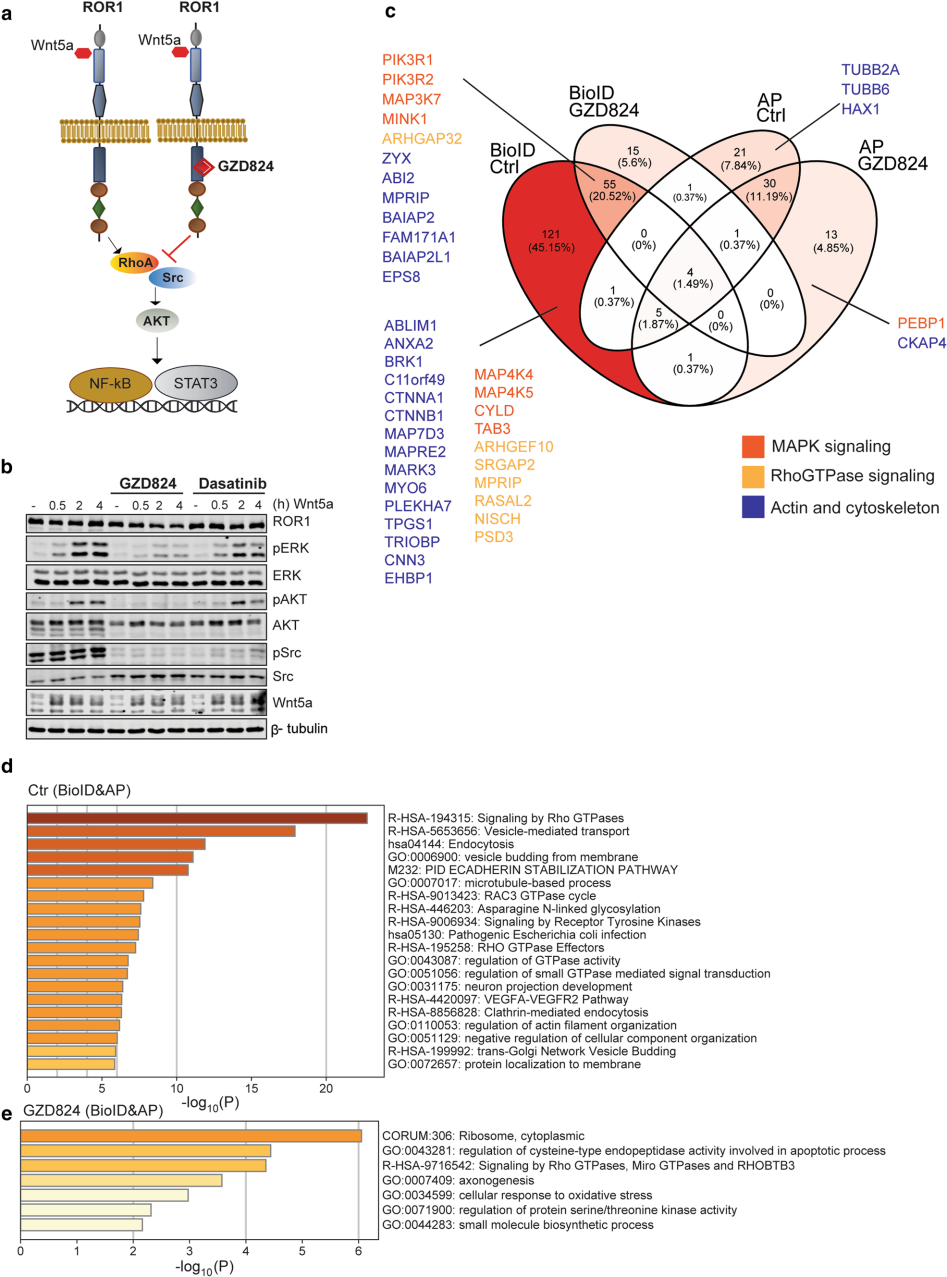
GZD824 inhibits downstream signaling of ROR1. **a** Schematic representation of GZD824 binding to ROR1 PK domain, which induces inhibition of its downstream PI3K/AKT, STAT3, and NF-κB signaling. **b** Immunoblot analysis of the BaF3-ROR1 cells left untreated of pre-treated with Src inhibitor dasatinib or GZD824 (1 µM) for 2 h before the addition of Wnt5a (100 ng/ml) as indicated. Downstream ROR1 signaling levels or pERK/ERK, pAKT/AKT, and pSrc/Src are shown. β-tubulin was used as a loading control. **c** Venn diagram showing the number of shared and specific interactors of ROR1 before and after GZD284 treatment as identified by AP-MS, BioID, or both. For each set of interactors, the protein-coding genes involved in MAPK signaling (red), Rho GTPases signaling (yellow), or actin/cytoskeleton signaling (blue) are indicated. **d**, **e** Bar graphs of the top non-redundant enriched ontology clusters from multiple functional annotation databases in ROR1 interactomes identified with both AP-MS and BioID when before (**d**) and after (**e**) GZD824 treatment. The color scale represents statistical significance

Next, we set to investigate how pharmacological targeting of ROR1 affects its interactome using GZD824 treatment followed by BioID and AP-MS (Fig. 5a, Table S4). GZD824 is a Bcr-Abl tyrosine kinase inhibitor that could bind other targets such as Src [45]. Pre-treatment of BaF3-ROR1 cells with GZD824 or dasatinib, a Src inhibitor that does not bind ROR1 [3] abolished Wnt5a-mediated pERK and pAKT activation only in GZD824-treated samples (Fig. 5b), suggesting that this inhibitor could act specifically downstream of Wnt5a-ROR1.

Treatment of ROR1-expressing Flp-In T-REx 293 T isogenic cell lines with GZD824 markedly impacted ROR1 interactome by reducing the number of interactors detected with BioID (70 proteins less compared to control) and AP-MS (53 proteins less compared to control). Interestingly, the number of ROR1 interacting proteins involved in Rho GTPase signaling and acting/cytoskeleton was decreased in GZD824-treated samples; however, many proteins involved in MAPK signaling were still able to interact with ROR1 in BioID (Fig. 5c). Consequently, enrichment analysis of the GZD824-treated interactomes from BioID and AP-MS did not lead to biological functions related to cytoskeleton or cell movement, and produced only one Rho GTPase-related term (Fig. 5d, e). Furthermore, the protein abundance of VANGL1, VANGL2, TAB1-3, DVL1-3, MYO6, PIK3R1, and PIK3R2 decreased after GZD824 treatment as measured with BioID, indicating a clear effect of the inhibitor on cytoskeleton dynamics, MAPK, Rho GTPase signaling, and Wnt/PCP pathway (Fig. S3).

Ultimately, we identified three proteins found solely in the untreated samples: catenin alpha-1 (CTNNA1), lipoma-preferred partner (LPP), and clathrin assembly lymphoid myeloid leukemia protein (PICALM) that have functions related to actin-binding, cell adhesion, and Rho GTPase activity, respectively. Taken together, these results indicate that GZD824 treatment affects ROR1 interactome by impairing its crosstalk with Rho GTPases signaling and its role in cytoskeleton modulation.

## Discussion

Increasing evidence emerging from functional and structural studies points toward Wnt-binding receptor pseudokinases having a key role in regulating functional processes in normal and pathological conditions, despite their lack of catalytic activity [3, 4]. Overexpressions of ROR1, ROR2, and PTK7 have been detected in numerous cancers. For instance, in hematological cancers the reactivation of ROR1 signaling in chronic lymphocytic leukemia (CLL), mantle cell lymphoma (MCL), and B-cell acute lymphoblastic leukemia (B-ALL) is linked to advanced disease stage and activation of AKT signaling in ROR1^-high^ positive cells [46–48]. ROR2 expression was also shown to activate AKT signaling in multiple myeloma [49], whereas PTK7 expression elicits anti-apoptotic effects in T cell acute lymphoblastic leukemia (T-ALL) [50].

Our proteomic analysis of BaF3 clones stably transfected with ROR1, ROR2, or PTK7 demonstrated similarities as well as differences in signaling specificities among these receptors. PCA indicated that BaF3-ROR1 and BaF3-ROR2 clones have a similar proteomic profile that is separated from the BaF3-PTK7 and parental BaF3 cells, which is expected from these closely related receptors from the same family. Among proteins with enhanced expression in BaF3-ROR1 and BaF3-ROR2 clones were Rack1, a downstream Rho GTPase previously linked to ROR2 signaling [51], and Abracl, which is involved in the regulation of actin and cytoskeleton dynamics processes [52]. Interestingly, a commonly upregulated protein in all BaF3 clones was Ide or insulysin, a multifunctional metallopeptidase protein involved in insulin degradation and insulin-mediated oncogenesis [53]. These novel results would indicate that ROR1, ROR2, and PTK7 could play a role in insulin turnover, and these findings should be further investigated.

Functionally, BaF3-ROR2 and BaF3-PTK7 showed enhanced migration compared to BaF3-ROR1 or parental BaF3 cells. However, BaF3-ROR1 clones survived better in the absence of growth factors compared to other BaF3 clones, clearly indicating a key role for ROR1 in mediating pro-survival signaling as previously documented [5]. Furthermore, higher expression of intracellular AKT and NF-κB was observed in BaF3-ROR1 compared to other clones, and this effect was more pronounced in BaF3-ROR1i cells that acquired growth-factor independence. Our proteomic analysis also found enriched expression of IQ motif containing GTPase activating protein 1 (Iqgap1) and Kindlin-3 (Fermt3) in our BaF3-ROR1 clones, which could contribute to activation of AKT/ERK/MEK pathway. Iqgap1 activates Rho-GTPase Cdc42 via binding to B-Raf, MEK, and ERK to induce MAPK activation in a range of tumors [54, 55]. Kindlins can activate integrin α5β1–AKT–mTOR pathway [56] and have been linked to oncogenic functions [57, 58]. Taken together, our BaF3 clones proved to be a useful model for the functional and biochemical characterization of stable overexpressed ROR1, ROR2, and PTK7 receptor pseudokinases, especially for avoiding the biases of transient transfections. However, several limitations are to be considered, such as the lymphoid origin of BaF3 cells and the lack of expression of other co-receptors or signaling molecules associated with the biology of ROR1/2 and PTK7 that are often co-expressed endogenously and may modulate their functional output. For instance, we could not address the mechanism of Wnt5a-mediated ROR1/2 hetero-dimerization in regulation of downstream signaling, which was previously shown to modulate leukemia chemotaxis and proliferation [32].

In the absence of catalytic activity, ROR1 and ROR2 can interact with other RTKs such as MET, epidermal growth-factor receptor (EGFR), and muscle-specific kinase (MuSK) or intracellular kinases such as Src and casein kinase CK1ε and become *trans*-phosphorylated [35, 59–62]. The majority of signaling events taking place at the receptor level were shown to be mediated by the Ser/Thr and Pro-rich regions of ROR1/2 that play a scaffolding role for downstream effectors [34–37]. Our C-terminal deletion analysis of ROR1/2 cytoplasmic region revealed that removing the distal Ser/Thr domain and the C-terminal tail resulted in overall lower receptor expression in both BaF3 stable clones as well as in 293T cells following transient transfections. Moreover, loss of ROR1 distal C-terminal region (Δ3 deletion) affected its downstream signaling, notably loss of AKT levels in BaF3-ROR1 Δ3 clone. This effect is most likely caused by the loss of putative adapters recruited to these sites that are involved in protein stabilization and proper folding, a mechanism previously observed with EGFR [63].

We have previously demonstrated that ROR1/2 PK domains are not able to bind ATP and/or cations to mediate a phosphotransfer reaction, thus being catalytically inactive [3]. Here, we show that neither the entire cytoplasmic region of ROR1 nor its C-terminal deletions can bind ATP and/or cations to mediate a phosphotransfer reaction. We also demonstrate that ROR1 is unable to undergo auto-phosphorylation even when its pseudokinase domain was fully mutated to an active kinase domain. This ascertains our previous results that ROR1 (and ROR2 as well) functions as a catalytically impaired pseudokinase, likely via scaffolding intracellular proteins that can induce intra- or inter-molecular changes responsible for signal initiation. This does not exclude a phosphorylation-dependent regulation of ROR1/2 signaling, as it is known that both receptors can undergo *trans*-phosphorylation [35, 59–62].

A versatile approach to identify the stable and transient interaction landscape of a target protein is the use of AP-MS and BioID methods, which enables the identification of protein complexes and their molecular network with relatively high accuracy [22]. Our BioID analysis identified a rich ROR1 PPIs network from previously known Wnt pathway (DVL, VANGL, β-catenin, and ROR2) and PI3K signaling (PI3K, GRB2, and MAPK) molecules to the unknown ROR1-interaction partners such as the general secretory (SEC23/24) pathway. On the other hand, AP-MS captured several protein complexes involving diverse effectors from chaperone to glycoproteome signaling. Most importantly, common ROR1 interacting proteins identified with both BioID and AP-MS included, among others, chaperone-binding proteins (CDC37 and DNAJA2), and damaged DNA-binding proteins (RPA1/2), which could extend ROR1-interaction network to pathways such as heat shock proteins and DNA repair. The interactomics insights gained here place ROR1 at the intersections of several signaling modules comprising of known but also previously unknown networks that should be further validated in ROR1-positive cells.

Targeting the extracellular domain of ROR1 with antibody-based pharmacological agents has been intensively investigated and several anti-ROR1 monoclonal antibodies have been generated [13, 64]. However, studies on the pharmacological modulation of ROR1 signaling using small molecule inhibitors are largely missing due to the lack of structural and biochemical studies of ROR1 cytoplasmic region. Previously, our chemical screens identified ponatinib and GZD824 as ROR1 small molecule binders and downstream signaling inhibitors, suggesting that the intracellular domain of ROR1 is suitable for pharmacological modulation [3]. When we applied BioID and AP-MS analysis to ROR1 PPIs’ network following GZD824 treatment, we observed a clear shift in the PPI landscape marked by the loss of Rho GTPase signaling and actin/cytoskeleton interactors. Although GZD824 is a pan-tyrosine kinase inhibitor that could bind to other tyrosine kinases downstream of ROR1 and thus, indirectly affecting its PPI network, our results showed that GZD824 treatment did not affect ROR1 MAPK interactome but only the Rho GTPase interactome. These results are of great pharmacological importance, as it would indicate that binding of a small molecule inhibitor to ROR1 would act distinctively on its downstream signaling modulation.

In conclusion, our results demonstrate the feasibility of targeting the catalytically impaired ATP-binding site of ROR1 to specifically modulate the downstream signaling in ROR1-expressing cells, which represents a valuable strategy for future ROR1-based targeted therapies.

## Supplementary Information

Below is the link to the electronic supplementary material.

Supplementary file1 (PDF 5368 KB)

Supplementary file2 (XLSX 47 KB)

## Data Availability

The original contributions presented in the study are included in the article/supplementary material, and further inquiries can be directed to the corresponding author.
