# Supplementary material for: New insights into the molecular mechanisms of ROR1, ROR2, and PTK7 signaling from the proteomics and pharmacological modulation of ROR1 interactome
Source: Cell Mol Life Sci. 2022 May 4;79(5):276. doi: 10.1007/s00018-022-04301-6 (PMC9064840; doi:10.1007/s00018-022-04301-6)
Supplement: Supplementary file 1 — Supplementary file1 (PDF 5368 KB) [file 18_2022_4301_MOESM1_ESM.pdf]

Supplementary Figure 1

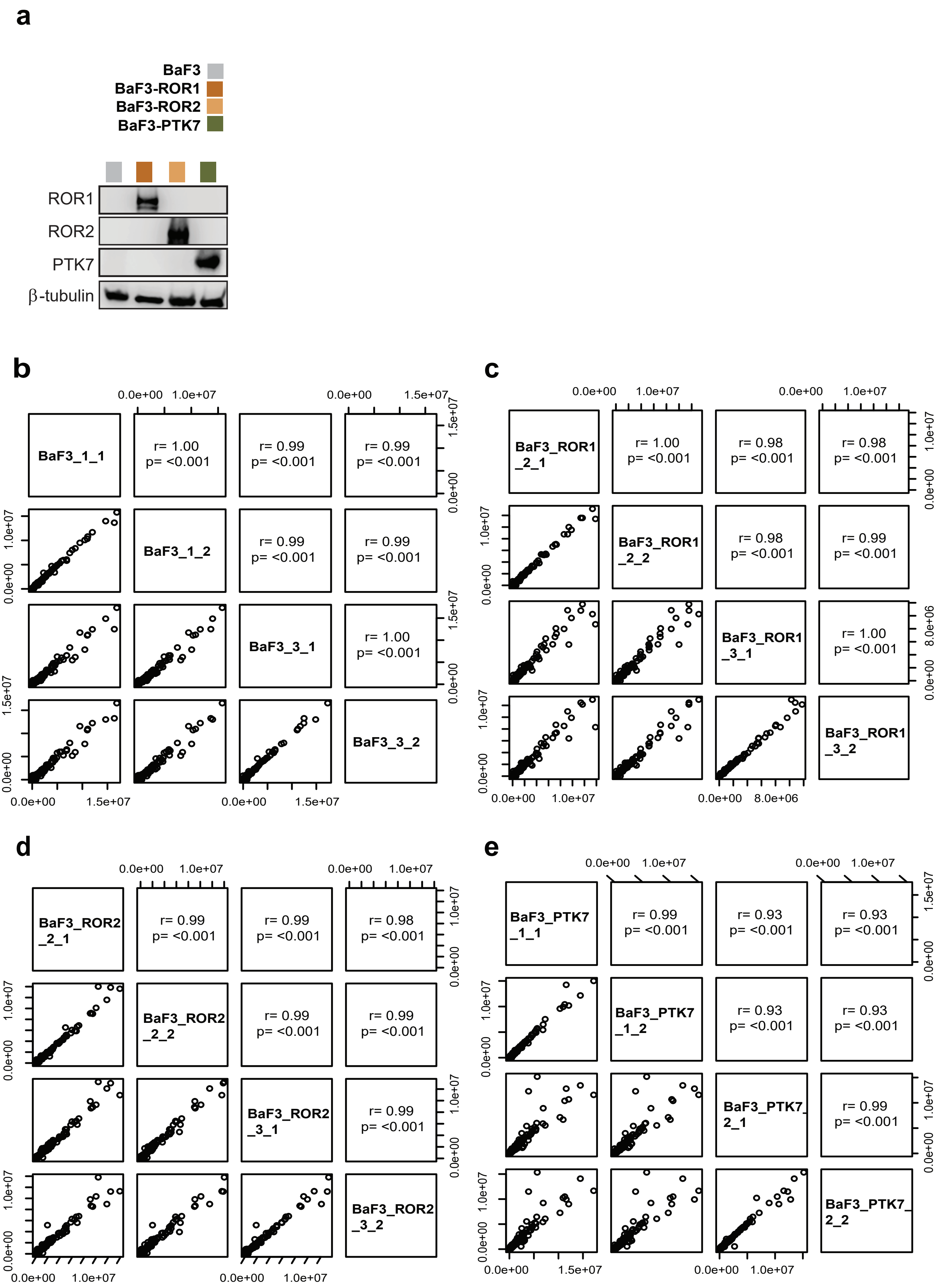

Fig. S1 Analysis of BaF3 clones stably expressing ROR1, ROR2 or PTK7.
a Immunoblot analysis of the cell lysates of the parental BaF3 and the BaF3 cells stably transfected with ROR1, ROR2 or PTK7. The three clones are correctly expressing the transfected receptor, while BaF3 parental cells do not express these receptors. β-tubulin was used as a loading control. **b-e** The biological replicates of each BaF3 clone positively correlate among each other in their proteomic profile. The Pearson´s coefficient (r) and p-values are reported.

Supplementary Figure 2

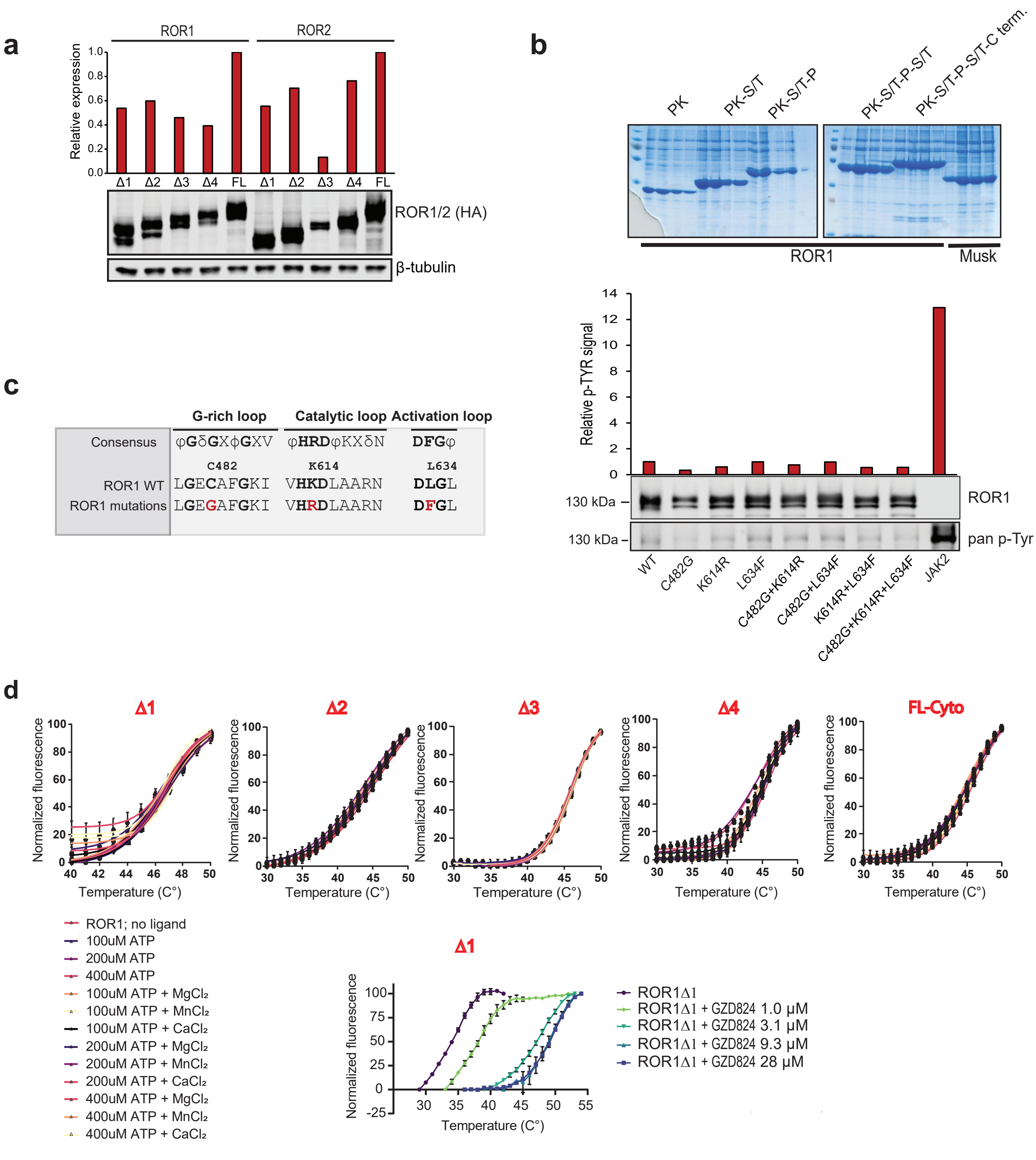

Fig. S2 Biochemical characterization of ROR1 (and ROR2).
a Immunoblot analysis showing the expression of the FL and deletion constructs of (HA-tagged) ROR1 and ROR2 in 293T cells. β-tubulin was used as a loading control. Bar chart depicts the normalized signal values (BaF3-ROR1/2 FL values set as 1). **b** SDS-PAGE analysis of the recombinant ROR1 proteins (cytoplasmic domains of ROR1) and the kinase domain of MuSK, showing the expression after Ni-NTA purification. **c** Left: Sequence alignment of ROR1 wild-type (WT) and the kinase activating mutations of the functionally important residues typically conserved among kinases but altered in ROR1. The consensus sequences are depicted above. Right: Immunoblot analysis of the ROR1 mutations transfected into BaF3 cells. Pan-tyrosine antibody (4G10, 05-321, Merck) was used to detect the tyrosine phosphorylation of ROR1 (HA). The bar chart shows the signal from the phosphorylated tyrosines in ROR1 (or JAK2), where the pTyr signal was normalized to ROR1 WT (set as 1). **d** DSF curves of the recombinant ROR1 C-terminal domains with no ligands and different concentrations of ATP and salts. The graphs depict the melting curves of triplicate samples; mean ± SD. Below, DSF curves of the ROR1 Δ1 construct showing increased thermal stability (Tm) following GZD824 binding.

Supplementary Figure 3

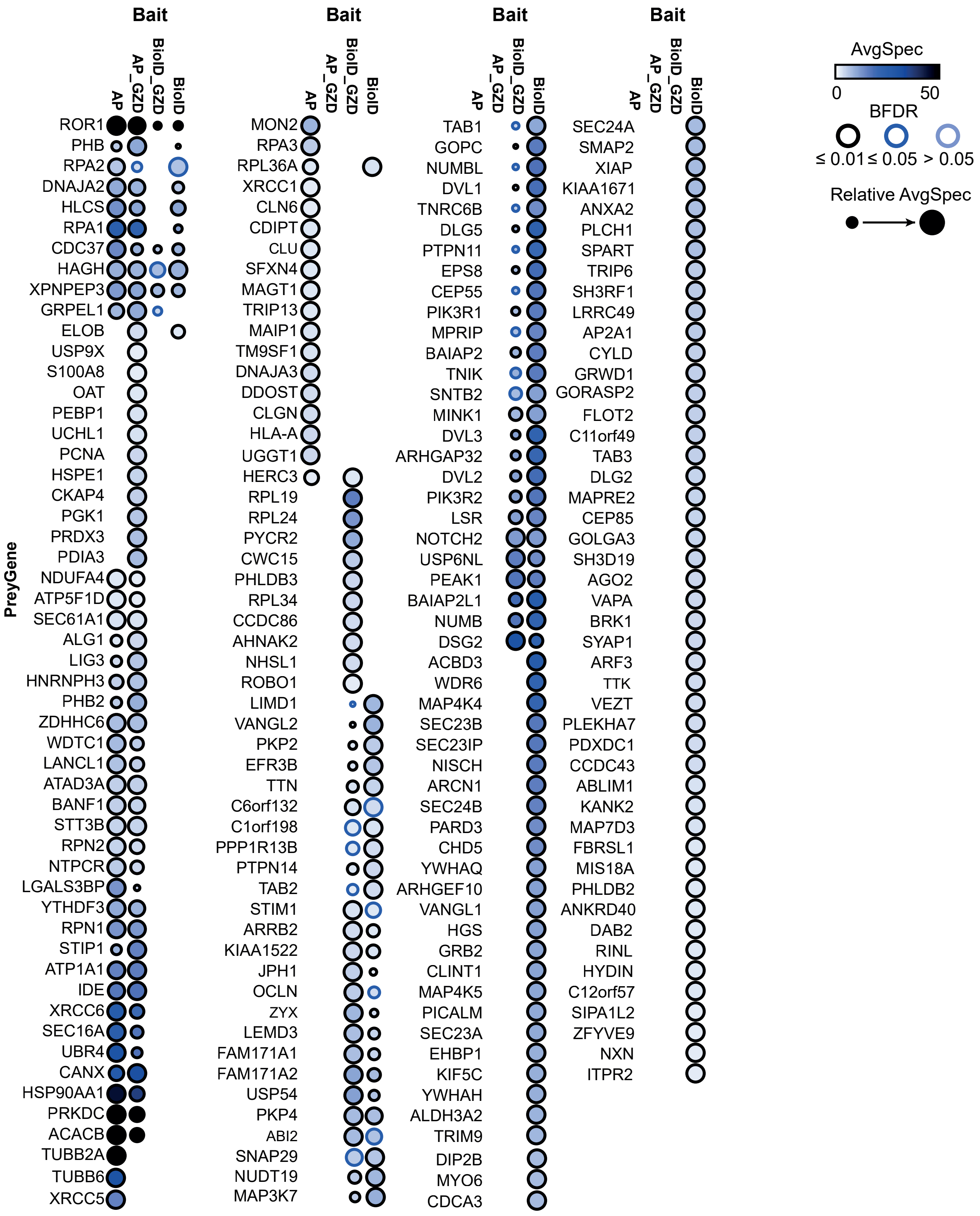

Fig. S3
BioID and AP-MS-based interactomes of ROR1-expressing Flp-In T-REx 293T cells untreated or treated with GZD824. The data are shown as dot plots reporting for each prey the average number of spectral counts (AvgSpec), the relative prey abundance towards the bait (relative abundance), and the Bayesian false discovery rate (BFDR) as confidence score.
